# An advanced genotyping tool to inspect grapevine variability: the Axiom^®^Vitis22K SNP array

**DOI:** 10.3389/fpls.2026.1771381

**Published:** 2026-03-18

**Authors:** Laura Costantini, Diego Micheletti, Paola Bettinelli, Andrea Minio, Lorenzo Spina, Daniela Nicolini, Michela Troggio, Dario Cantù, Silvia Vezzulli, Luca Bianco

**Affiliations:** 1 Research and Innovation Centre, Fondazione Edmund Mach, San Michele all'Adige, Trento, Italy; 2 EURAC, Institute for Biomedicine, Bolzano, Italy; 3 Department of Viticulture and Enology, University of California, Davis, Davis, CA, United States; 4 University School for Advanced Studies Istituto Universitario di Studi Superiori (IUSS) Pavia, Pavia, Italy; 5 Genome Center, University of California, Davis, Davis, CA, United States

**Keywords:** Genetic diversity, genome-wide analysis, high-throughput genotyping, marker/genome assisted breeding, *Vitis* spp.

## Abstract

Grapevine is one of the most relevant fruit crops worldwide, owing to its extensive distribution and considerable socio-economic significance. While the cultivated Eurasian species *Vitis vinifera* dominates global grape production, wild *Vitis* species from Asia and North America constitute essential genetic reservoirs, offering allelic diversity associated with tolerance or resistance to several biotic and abiotic stresses. Genotyping is a key tool in grapevine genetics, as it enables the assessment of genetic diversity, the elucidation of the molecular basis of agronomic and adaptive traits, and the implementation of marker-assisted selection. SNP (single nucleotide polymorphism) arrays provide an efficient genotyping tool, combining high-throughput capability, cost-effectiveness, and dense genome-wide marker coverage. Here, we report the development and validation of the Axiom^®^Vitis22K SNP array, implemented within a broader multi-species 70K SNP platform. The array includes informative SNPs from the GrapeReSeq 18K Vitis genotyping chip, SNPs and small InDels (insertions and deletions) putatively associated with phenotypic traits from literature, and novel SNPs obtained from the resequencing of 12 samples representing seven different grapevine species. Validation was performed by genotyping 144 genotypes from two diversity panels. Genotyping data were processed with the Axiom Analysis Suite and the newly developed AxioSAFE pipeline revealing a total of 10,314 robust polymorphic markers, together with 13 manually curated variants. These markers were successfully employed to study the genetic diversity and genetic relationships within the sample panels. Integration of genotypic and phenotypic data also enabled the validation of candidate SNPs associated with target traits such as flower sex type, seed content, berry color and taste. Overall, the results demonstrate the potential of the Vitis22K array to support large-scale genetic studies and breeding programs in grapevine.

## 1 Introduction

Among the various types of mutations, Single Nucleotide Polymorphisms (SNPs) are the most ubiquitous form of sequence variation in plant genomes (Brookes, 1999; Ganal et al., 2009), serving as indispensable molecular markers for genetic studies. The applications of SNPs in crop genetics have been extensively reviewed by Batley and Edwards (2007). In recent decades, we have moved from numerous low-throughput (including non-sequence-based) genotyping techniques, through mid-throughput methods, to high-throughput approaches (Ragoussis, 2006). Over the past few years, we have witnessed the rapid expansion of ultra-high throughput genotyping techniques, driven by continuous advances in sequencing strategies (Thomson, 2014).

Whole-Genome Sequencing (WGS) provides comprehensive information by sequencing the entire plant genome, detecting all sequence variants, including SNPs and other structural variants such as insertions and deletions. Although sequencing throughput is steadily increasing and costs are decreasing, WGS remains the most expensive approach on a per-sample basis (Goodwin et al., 2016) and requires substantial computational resources and bioinformatic skills for the downstream analysis. An attractive alternative for high-throughput population studies is Genotyping-by-Sequencing (GBS), which generates high-density markers from a reduced representation of the genome at a lower cost than WGS (Elshire et al., 2011; Zhao et al., 2025); however, it still requires advanced bioinformatic expertise and is prone to missing data resulting from uneven coverage across samples.

SNP genotyping arrays are a highly parallelized technology that utilizes probe hybridization and a fluorescence detection system to simultaneously genotype thousands of predetermined SNP loci across large sample sets (You et al., 2018). Although probe design must be defined in advance, leading to well-known issues like the ascertainment bias (Lachance and Tishkoff, 2013), SNP arrays have been successfully employed in several crop species (Ganal et al., 2012; You et al., 2018) and currently offer competitive per-sample costs in large-scale studies.

Among the most cultivated, highly heterozygous tree species, grapevine stands out due to its widespread distribution and socio-economic importance (OIV (International Organisation of Vine and Wine), 2025). In terms of SNP-based genotyping tools, several efforts have been carried out in order to support dense genetic mapping (e.g. Vervalle et al., 2022), germplasm characterization (e.g. Žulj Mihaljević et al., 2020), and marker-assisted breeding (Luca et al., 2024).

Here we describe the development of a novel Axiom^®^Vitis22K SNP array, which is part of a larger, multi-species 70K SNP array also including chestnut, hazelnut and black walnut (Bianco et al., 2023). The Axiom^®^Vitis22K SNP array includes 21,874 SNPs evenly distributed across the genome of which 285 SNPs are putatively associated with traits of interest and 9,286 are from wild species. A total of 11,701 robust SNPs from the Illumina GrapeReSeq 18K Vitis chip (Le Paslier et al., 2013) were included to ensure compatibility with previous genotyping studies.

## 2 Materials and methods

### 2.1 SNP selection strategy

The list of variants (SNPs/InDels) tiled in the newly designed Axiom^®^Vitis22K SNP array was obtained by combining three different sources: i) robust SNPs already present in the previous GrapeReSeq 18K Vitis genotyping chip, ii) SNPs and InDels putatively associated with phenotypic traits identified through a literature survey, and iii) newly identified SNPs derived from the resequencing of 12 samples representing seven grapevine species.

Markers were retrieved from the previous GrapeReSeq 18K Vitis genotyping chip (Le Paslier et al., 2013) for inclusion in the new Axiom^®^Vitis22K SNP array, comprising both *V. vinifera* and non*-vinifera* species. All the SNPs from non*-vinifera* species (*V. aestivalis, V. berlandieri, V. cinerea, V. labrusca, V. lincecumii* and *M. rotundifolia*) were recovered without further selection, as insufficient genotyping studies were available to allow an appropriate filtering. In contrast, the SNPs from *V. vinifera* were selected among the 13,560 available markers based on the following partially overlapping criteria to maximize informativeness: a) SNPs included in the genotyping set adopted by the *Vitis* International Variety Catalogue (*V*IVC, Röckel et al., 2025^1^), b) SNPs shared across three genetic maps and representative SNPs from the ‘Rhine Riesling’ × ‘Cabernet Sauvignon’ map in Vervalle et al. (2022), c) representative SNPs from the ‘Merzling’ × ‘Teroldego’ map and all the SNPs on chromosome 14 of the same map (Bettinelli et al., 2023), d) SNPs with a minor allele frequency (MAF) greater than 0.45 in Laucou et al. (2018), e) SNPs consistently showing genotyping success and polymorphism rate greater than 0.40 in at least seven out of nine datasets examined (Emanuelli et al., 2013; De Lorenzis et al., 2015, 2019; Laucou et al., 2018; Crespan et al., 2020, 2021; Duchêne et al., 2020; Raimondi et al., 2020; D’Onofrio et al., 2021), f) SNPs with a polymorphism rate greater than 0.40 in the rootstock collection analyzed by Bianchi et al. (2020), g) SNPs included in the panel of highly informative markers suggested by Mercati et al. (2016), h) plastidial SNPs from Raimondi et al. (2020).

A total number of 289 SNPs or small InDels were selected based on reported associations with phenotypic traits of interest as described in the literature available up to July 2023 (**Supplementary Table 1**). For each polymorphism, genomic coordinates were reported on the positive strand of either the PN40024 12X.v2 reference genome assembly^2^ or the phased diploid Cabernet Sauvignon genome assembly (CabSauv08 v.1.1^3^). The presence of each SNP marker on the GrapeReSeq 18K Vitis chip was assessed according to its position on the PN40024 12X.v2 assembly, as reported in Vervalle et al. (2022). If so, the original name in the GrapeReSeq 18K Vitis chip was adopted as primary SNP ID in the new Axiom^®^Vitis22K SNP array. Information about the polymorphism type (i.e. intergenic or intragenic and, in the latter case, position within the gene), the gene eventually involved, and the genotype(s) showing the polymorphism was retrieved from the original publication. The affected phenotype was expressed with the name of the variable as reported in the original publication, as well as according to Vitis Ontology V2^4^, where feasible. The effect of the alternative allele on the associated phenotype was classified, when possible, as gain or loss of function relative to the reference allele.

Genomic resequencing data of 12 samples from seven grapevine species were used to increase the diversity represented in the array, with SNP discovery based on the download and re-analysis of high-quality publicly available datasets. Specifically, four samples belonged to *V. vinifera* ssp*. sylvestris* (Massonnet et al., 2020), two to *V. vinifera* ssp. *sativa* (Massonnet et al., 2020; Zou et al., 2020), and one sample each to *M. rotundifolia* (Cochetel et al., 2021), *V. amurensis* (Wang et al., 2021), *V. arizonica* (Massonnet et al., 2020), *V. piazeskii* (Massonnet et al., 2025), *V. romanetii* (Zou et al., 2021) and *V. rupestris* (Zou et al., 2020). One sample was sequenced with PacBio, while the remaining 11 were sequenced with Illumina platform (**Supplementary Table 2**). All the Illumina reads were aligned on the PN40024 12X.v2 reference genome using BWA mem (Li, 2013), whereas PacBio reads were aligned using minimap2 (Li, 2021). Variant calling was performed using GATK v.4.0.12.0 (Poplin et al., 2017) for Illumina datasets and Clairvoyante for PacBio data (Luo et al., 2019; https://github.com/aquaskyline/Clairvoyante). After merging all the identified variants in a single file, SNPs located within 35 nucleotides of another polymorphism were flagged, using a custom script, to prevent probe interference. Filtering criteria included removal of low-quality variants (QUAL < 100), insertions and deletions, multiallelic SNPs, positions with depth (DP) < 8, SNPs with more than 20% of missing data, and C/G and A/T polymorphisms. Filtering was carried out using bcftools (Danecek et al., 2021), vcftools (Danecek et al., 2011) or custom scripts.

All variants included in the array were also aligned to the PN40024.T2T (v5) reference genome (Shi et al., 2023) using BLAST. SNPs were assigned preferentially to their positions on the reference (REF) chromosomes; coordinates on alternative (ALT) chromosomes were retained only when the highest-scoring BLAST hit corresponded to an ALT sequence. Plastidial markers were assigned to the chloroplast genome rather than to Chr00, which in PN40024.T2T (v5) also contains chloroplast-derived sequences.

### 2.2 SNP final choice for the Axiom^®^Vitis22K SNP array

**Figure 1:**
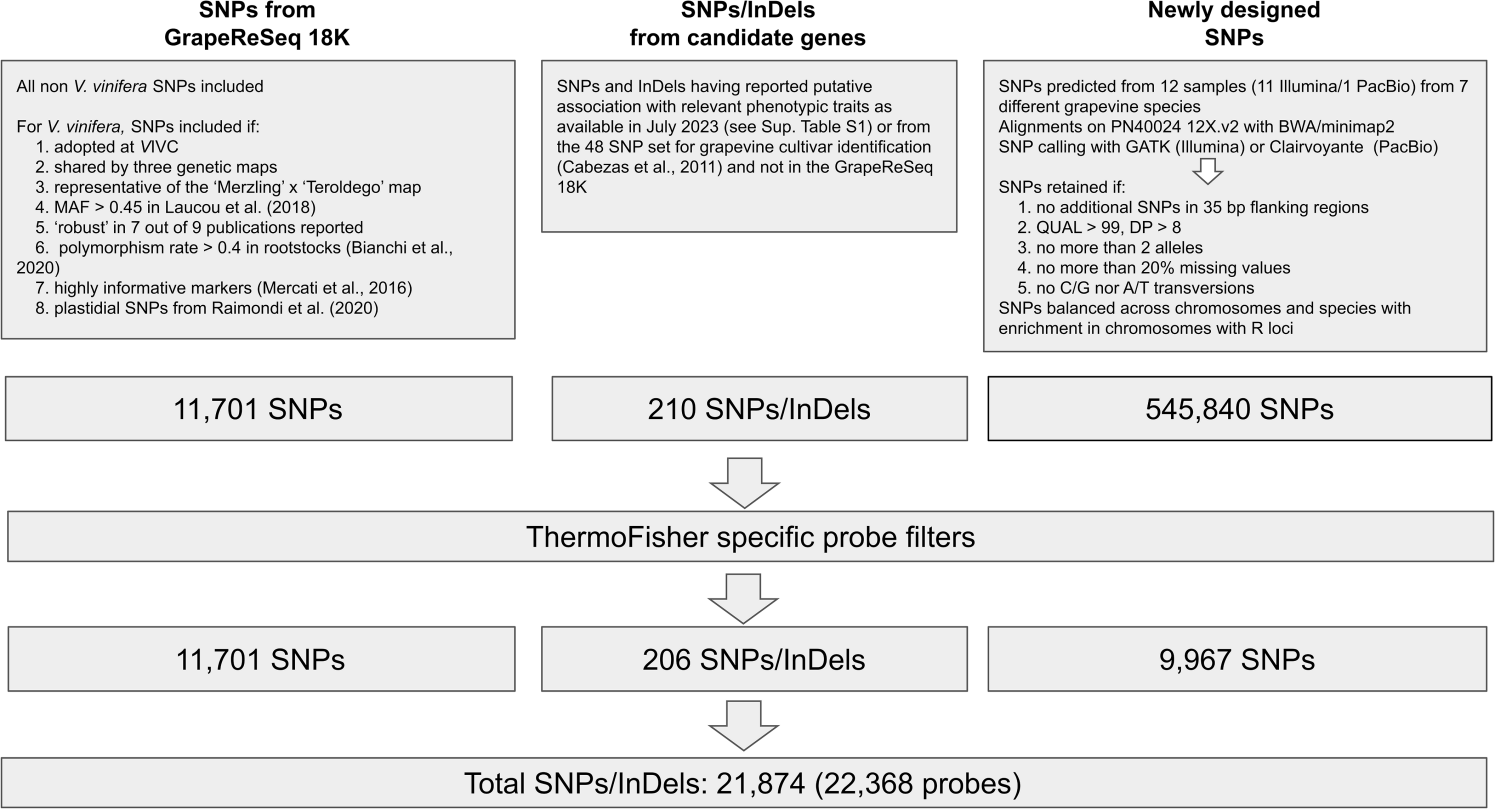
Strategy for variant selection and final number of SNPs and InDels tiled in the Axiom^®^Vitis22K SNParray.

A flanking region of 50 nt up-stream and down-stream for all the SNPs was submitted to ThermoFisher’s proprietary scoring system. For categories (as defined at the beginning of Section 2.1) i. and iii. only SNPS with at least one probe flagged as ‘Recommended’ were retained. For category ii. two probes (one on each side of the variant) were included regardless of recommendation score to maximize capture probability. Newly identified SNPs were used to complete the final list of 22K SNPs, ensuring a targeted distribution across species and chromosomes. Specifically, 40 biallelic SNPs per chromosome were selected for each wild species and 130 SNPs per chromosome for *V. vinifera*. The chromosomes containing known Resistance (R)loci in the wild species (Vezzulli et al., 2022) were further enriched with 60 additional SNPs. A schematic representation of the strategy for the selection of variants to be included in the array is reported in **Figure 1**.

### 2.3 Pilot SNP array hybridization on two diversity panels

A pilot hybridization experiment was carried out on two *ad hoc* created panels: i) a grapevine breeding parental panel (BPP) comprising 95 genotypes, including two somatic variants, currently used in breeding programs at FEM (Fondazione Edmund Mach), and ii) a grapevine flower sex diversity panel (FSP) consisting of 48 genotypes (**Supplementary Table 3**). All plant material derived from the FEM germplasm collection (Bettinelli et al., 2024). Trueness-to-type was previously checked by genotyping with the universal set of nine microsatellite markers (Maul et al., 2012). When available, the primary accession names adopted at *V*IVC were retrieved. Four traits of interest (flower sex, seed development, berry skin color and taste) were selected for SNP validation, and their corresponding phenotypes were assigned to each genotype by integrating our visual observations, unpublished data, and information available from the *V*IVC or the literature. Flower sex was classified as female, hermaphrodite, or male, while seed development was categorized as complete or none. Berry skin color was annotated according to the OIV descriptor 225^5^. Finally, berry taste was assigned to the following classes, using the *V*IVC descriptors as a reference: foxy, herbaceous, muscat, muscat-like aromatic and none. When feasible, pedigree information was recovered from the *V*IVC or from FEM breeding records and was additionally inferred from SNP data. Twenty-seven of the genotypes included in the BPP had been previously genotyped with the GrapeReSeq 18K Vitis chip (**Supplementary Table 3**). Genomic DNA was extracted from 100 mg fresh young green leaves with the QIAGEN ^®^ DNeasy Plant Mini Kit according to the manufacturer’s instructions.

Genotyping of the genomic DNA using Axiom technology was performed at the FEM Sequencing and Genotyping Platform using the Axiom^®^Vitis22K SNP array on the GeneTitan™ MC Instrument following the Axiom™ 2.0 Assay 96-Array Format Manual Workflow. For the previous hybridization on the GrapeReSeq 18K Vitis chip, 50 nanograms of DNA were employed following quantification with the NanoDrop™ Spectrophotometer 2000 (Thermo Scientific™). GenomeStudio v2011.1 (Illumina Inc., San Diego, CA, USA) was used for clustering raw SNP data by applying a GenTrain threshold of 0.70 and a minimum call rate of 95% to discard poorly performing samples.

### 2.4 Pre- and post-processing SNP analysis

Genotyping data from the Axiom^®^Vitis22K SNP array were processed using the Axiom Analysis Suite (AxAS) software following the “Best Practices Workflow”. All default parameters were used, except for the quality control (QC) call rate, which was lowered from 0.97 to 0.85 to accommodate the presence of several *Vitis* species in the analyzed panel, which results in additional missing calls caused by the species-specific design of certain SNPs.

Preliminary SNP filtering was performed using AxioSAFE (Spina et al., 2026), which takes as input the genotype matrix exported from AxAS through the “Genotype - Export” option in the “Probeset Summary Tab” of the AxAS project window. AxioSAFE was run with default parameters to perform ploidy checks, filtering based on the clustering metrics (‘filterm’), removal of additional clusters (‘filterc’), and pedigree analysis for Parent-Offspring -PO- and Parent-Parent-Offspring -PPO- relationships. Pedigree analysis using the *duos* and *trios* commands was conducted iteratively to minimize Mendelian segregation errors and incorporate all known PO relationships. In the initial iteration, analyses were performed using default parameter settings. In the subsequent iteration, the maximum allowable error rate for the *duos* command was relaxed to capture all expected true PO pairs. This adjustment ensured the inclusion of all parental individuals in the *trios* analysis. Following each AxioSAFE run, SNPs exhibiting more than 3% Mendelian segregation errors were excluded using the *filterp* function. The phasing step was omitted due to the limited number of individuals, low number of expected PPO relationships, and high heterogeneity of the dataset, which represents known limitations for accurate haplotype inference using probabilistic software such as SHAPEIT (Hofmeister et al., 2023) implemented in AxioSAFE.

Principal component analysis (PCA) was performed to study genotype diversity and relatedness among genotypes. The first PCA was conducted with PLINK v1.9 (Purcell et al., 2007) after filtering markers with a minor allele frequency (MAF) lower than 0.05. A second PCA was performed in R (version 4.3.3) using FactoMineR package (Lê et al., 2008) on four pairwise pedigree parameters of the ‘duos’ analysis derived from AxioSAFE: percentage of Mendelian error (duo_errors_perc), number of directional heterozygous mismatches (het_mismatch_A1A2, het_mismatch_A2A1), and percentage of heterozygous mismatches (perc_mismatches). The factoextra R package (Kassambara and Mundt, 2020) was used for the extraction and visualization of multivariate analysis results.

### 2.5 Technology comparison: reproducibility test

To assess reproducibility between technologies, SNPs common to both the GrapeReSeq 18K Vitis chip and the Axiom^®^Vitis22K SNP array were examined. A subset of 27 genotypes previously genotyped with the GrapeReSeq chip was re-genotyped with the Axiom platform (**Supplementary Table 3**). For each sample, concordance between heterozygous and homozygous genotype calls was assessed. SNPs with missing values in either platform were discarded. The reproducibility analysis was repeated after curation of the Axiom^®^Vitis22K SNP array dataset with the pipeline AxioSAFE (Spina et al., 2026) with default parameters. In this case, SNPs flagged as “PASS” or “MONO_HIGH_RESOLUTION” (i.e. SNPs showing only monomorphic genotypes) were considered as valid loci.

### 2.6 PCR validation

To validate SNPs associated with key traits included in the Axiom^®^Vitis22K SNP array, all genotypes of the BPP and FSP panels were also genotyped using PCR-based markers for flower sex (VVIB23) and berry color (VvMybA1), according to the original publications (Lijavetzky et al., 2006; Battilana et al., 2013).

VVIB23 PCR products were separated by capillary electrophoresis on a ABI 3130xl Genetic Analyzer (Life Technologies, Foster City, CA, USA) and scored with GeneMarker software v3.0 (SoftGenetics, State College, PA, USA), using the GeneScan 500 LIZ size standard as an internal ladder (Life Technologies, Foster City, CA, USA).

For berry color prediction, primers a (5’-AAAAAGGGGGGCAATGTAGGGACCC-3’) and d3 (5’-CCTGCAGCTTTTTCGGCATCT-3’) were used to amplify the VvmybA1^ITA^ allele, while primers b (5’-GGACGTTAAAAAATGGTTGCACGTG-3’) and d3 to amplify the VvmybA1^AFL^ allele, respectively. PCR conditions consisted of an initial denaturation step of 95 °C for 5 min, followed by 35 cycles of 94, 65 and 72 °C for 1 min each, and a final extension step of 72 °C for 10 min. Amplification products were separated by electrophoresis in 1.5% agarose gels.

## 3 Results

### 3.1 Overall SNP statistics

Overall, 22,368 probes representing 21,874 variants were included in the array. The number of probes is higher than the number of SNPs to target both sides of essential variants and to capture A/T and C/G transversions or indels. Of these 21,874 variants, 11,701 come from the GrapeReSeq 18K Vitis chip -including 89 markers associated with phenotypic traits-, 9,967 variants derive from the sequencing of 12 different *Vitis* species and clones, and the remaining markers were included because linked to phenotypic traits as detailed in **Supplementary Table 1** (**Figure 1**). Of the markers from the GrapeReSeq 18K Vitis genotyping chip 7,201 originated from *V. vinifera* and 4,509 from non-*vinifera* species (**Supplementary Table 4**). Analysis using the Axiom Analysis Suite (AxAS) v.5.4 Best Practice Workflow revealed a 10,219 PolyHighResolution, 2,807 MonoHighResolution, 2,107 NoMinorHom, 3,701 Other, 1,678 OTV, and 944 CallRateBelowThreshold. The remaining 912 ProbeSetIDs showed an excessive variance either in Size or Contrast for at least one genotypic class.

AxioSAFE data curation identified, as expected, no polyploid individuals. Two duplicate pairs were detected, namely ‘Sangiovese’ and its somatic variant ‘Sangiovese seedless’ (6 differences excluding the NC), and two individuals previously considered full-siblings (Kozma 1-1–09 and Kozma 1-1-93), which differed by only two markers excluding the NC. Overall, 10,314 SNPs were classified as PASS by AxioSAFE and were considered for further analysis (5,978 from the GrapeReSeq 18K Vitis chip, 110 specifically linked to phenotypic traits and 4,226 from resequencing). Of the 8,313 PolyHighResolution markers validated as ‘PASS’ by AxioSafe, 6,375 were identified as polymorphic in *V. vinifera*. Among the 2,001 NoMinorHom markers, 743 were derived from *V. vinifera* - specifically 455 from ssp. *sylvestris* - highlighting a significant proportion of species-specific or private alleles that reflect the distinct genetic backgrounds of the different species. All the details of the SNP classification are reported in **Supplementary Table 5**. An additional 13 polymorphisms were manually recovered for their putative predictive value: six chloroplastic SNPs, four candidate SNPs for flower sex, two for berry taste, and one InDel for berry color (with * in **Supplementary Table 6**).

**Figure 2:**
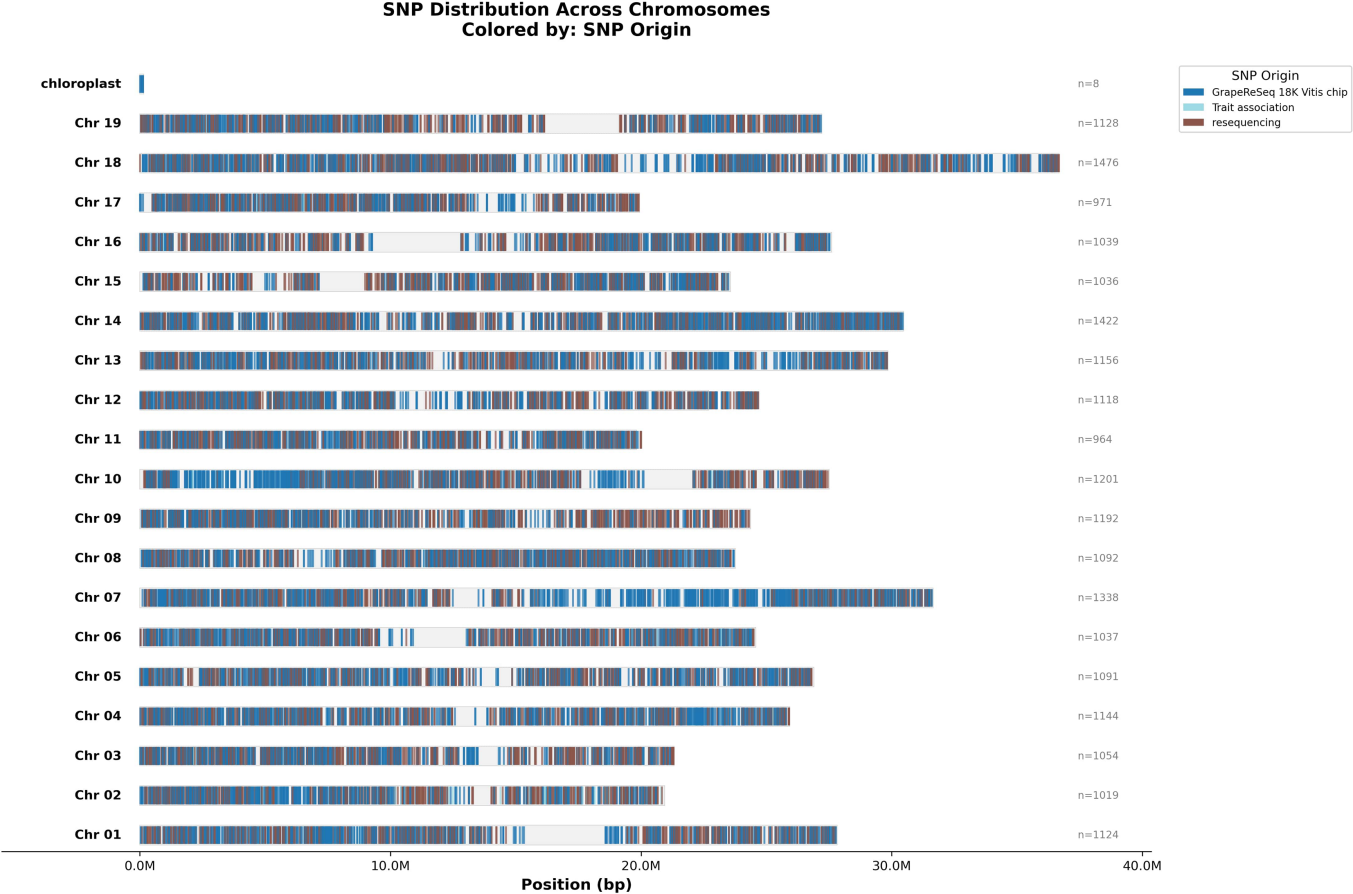
Distribution of the 21,610 variants of the Axiom^®^Vitis22K SNParray that have a unique position on the PN40024.T2T (v5) genome.

Among the retained markers, 21,610 variants were mapped on PN40024.T2T (v5), 55 were not positioned or in unanchored regions, and 209 were mapped on the ALT chromosomes. Their distribution across chromosomes showed uniform coverage. Centromeric regions were found to be markedly less enriched in markers than the adjacent chromosomal regions (**Figure 2**).

The average call rate among the 144 samples genotyped was 96.7% with a minimum of 93.1% in ‘Cinerea Arnold’ (*V. cinerea*). In the *V. vinifera* ssp. *sativa* the average call rate was 97.5% (**Supplementary Table 3**). The lower values were observed in non-*vinifera* samples. This results demonstrates that the PN40024-based probe design successfully hybridized across the genus without significant clustering issues due to additional variability affecting the probes and not accounted for in the design stage. The average observed heterozygosity was 22.8% with a minimum of 5.8% in Coia 05 and a maximum of 33.0% in ‘Merzling’. As expected according to SNPs origin (almost half of the SNPs in the array come from *V. vinifera*) the less heterozygous species were the wild species while the two subspecies of *V. vinifera* showed a higher level. The 56 hybrids showed a highly variable level and the higher values were observed in hybrids involving *V. vinifera* ssp*. sativa* (**Table 1**).

**Table 1:** Observed heterozygosity metrics across *Vitis* species.

Species	No. of accession	Average obsHet	Min obsHet	Max obsHet	Average calls
*Vitis champinii*	1	0.15	0.15	0.15	10,103
*Vitis cinerea*	1	0.08	0.08	0.08	9,903
*Vitis hybrid*	56	0.26	0.09	0.33	10,281
*Vitis labrusca*	4	0.09	0.06	0.15	10,104
*Vitis longii*	1	0.08	0.08	0.08	10,195
*Vitis riparia*	4	0.08	0.08	0.08	10,115
*Vitis rupestris*	1	0.09	0.09	0.09	10,171
*Vitis vinifera* ssp. *sylvestris*	22	0.2	0.11	0.24	10,267
*Vitis vinifera* ssp. *sativa*	49	0.25	0.2	0.3	10,299
*Vitis vulpina*	1	0.1	0.1	0.1	10,083

Two samples of *V. vinifera* ssp. *sylvestris* (Coste del Cese 1 and Pinnia 2) were excluded due to misclassification. Summary of accession numbers, average, minimum, and maximum observed heterozygosity (ObsHet), and average genotyping call counts for each *Vitis* species.

### 3.2 Technology comparison

Of the 11,701 SNPs present in the GrapeReSeq 18K Vitis chip, 11,697 were also included in the newly designed Axiom^®^Vitis22K SNP array. In fact, 4 SNPs (IDs: VVI_10113, VVI_10992, VVI_9227, and VVI_9920), although selected for incorporation, were not tiled in the previous GrapeReSeq 18K Vitis chip due to technical reasons. A total of 27 genotypes were genotyped with both technologies and a comparison of heterozygous versus homozygous calls, excluding SNPs with missing values from the analysis, was performed. The concordance rate ranged from 84.51% (9,885 SNPs) for Coia 01 to 93.69% (10,959 SNPs) for ‘Muscat Ottonel’. The average concordance between the two technologies was 90.72%, ranging from 89.43% in *Vitis* hybrids (VH) to 93.32% in *V. vinifera* (VV) samples on a total of 10,611 SNPs) (**Supplementary Table 7A**). The same comparison was performed after data curation of the Axiom dataset with the pipeline AxioSAFE considering “PASS” and “MONO_HIGH_RESOLUTION” variants. In this case, a total of 6,692 SNPs were present in both datasets and the het/hom concordance rate ranged from 94.75% for ‘Tamiami’ to 99.22% for ‘Muscat Ottonel’. The average concordance between the two technologies was 97.91% (ranging from 97.36% in VH to 99.00% in VV samples) (see **Supplementary Table 7B**).

### 3.3 Scouting the genetic diversity for breeding purposes

To enable a more accurate interpretation of the diversity observed in Vitis hybrids, the genetic background of each accession was considered in terms of species represented, in addition to taxonomic classification (**Supplementary Table 3**). The following classes were defined: i) Vitis complex hybrid (including M. rotundifolia and V. vinifera), ii) Vitis hybrid with V. vinifera, iii) Vitis hybrid without V. vinifera, iv) Vitis complex hybrid × V. vinifera ssp. sativa, and v) Vitis hybrid × V. vinifera ssp. sativa.

**Figure 3:**
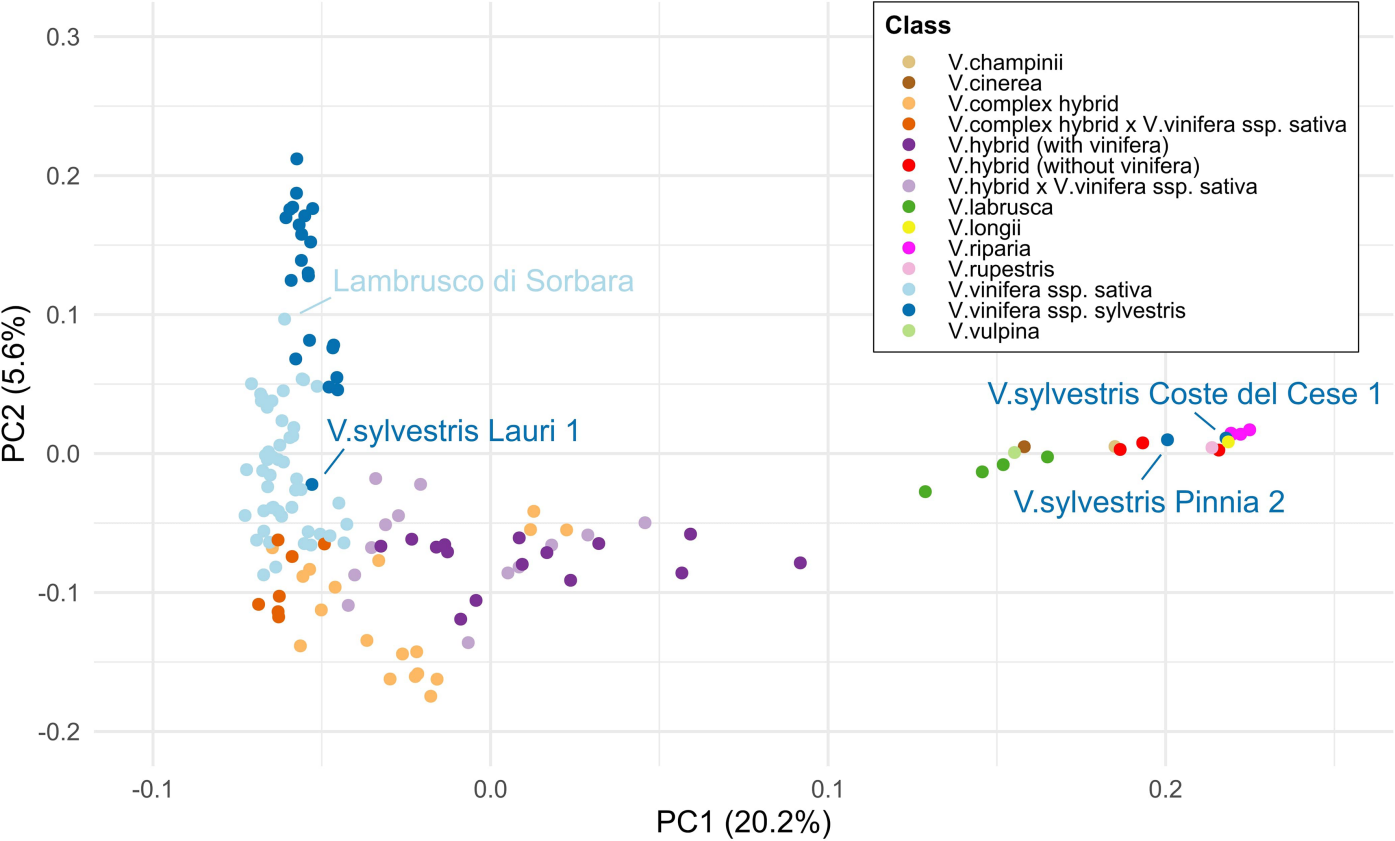
Principal component analysis (PCA) based on SNP data illustrating the genetic diversity of the studied panel with colors indicating the taxonomic classes defined in this study. The first two principal components (PC1 and PC2) explain 20.2% and 5.6% of the total variance, respectively. Labeled genotypes were found to be misclassified. The clustering pattern highlights the genetic distinction between wild and cultivated *Vitis* groups and their related hybrids (PC1), as well as between the *V. vinifera* ssp. *sativa* and *sylvestris* (PC2).

Upon PCA analysis, PC1 explained 20.15% while PC2 explained 5.55% of the genetic variability. PC2 separated *V. vinifera* subspecies, *sativa* and *sylvestris*, while PC1 separated *V. vinifera* from *Vitis* wild species (non-*vinifera*), passing through the *Vitis* hybrid compartment (**Figure 3**). Among the *Vitis* hybrids with ssp. *sativa* in their background, ‘Seyval Blanc’ was the most distant in terms of pure *Vitis* species, followed by another Seyve Villard hybrid, ‘Villard Blanc’. Along PC1, ‘Tamiami’ was the *Vitis* hybrid × *Vitis vinifera* ssp. *sativa* cross closest to the pure *Vitis* species. Moreover, *V. sylvestris* Lauri 1 (female) clustered with the group of *Vitis vinifera* ssp. *sativa*, while *V. sylvestris* Coste del Cese 1 (female) and Pinnia 2 (male) clustered with *Vitis* wild species. Finally, *V. vinifera* ssp. *sativa* ‘Lambrusco di Sorbara’ fell within the group of *V. vinifera* ssp. *sylvestris.*

Genetic relationships within the panel were explored based on the AxioSAFE PO and PPO (‘duos’ and ‘trios’) results. The PO error threshold was set to the maximum error observed in a known PO relationship (0.92%). Using this parameter, a total of 114 PO and 38 PPO relationships were identified by AxioSAFE. All expected PO relationships were confirmed, except for two cases: i) for ‘Petra’ the most probable *V. vinifera* parent was ‘Gewuerztraminer’ instead of ‘Pinot Noir’ ii) for the hybrid IV 8/1 ‘Petra’ was not detected as a possible parent (**Supplementary Table 3**). Moreover, ‘Manzoni Bianco’ showed PO relationships not only with its known parents (duo error 0.02% and 0.03%) but also with ‘Gewuerztraminer’ (duo error 0.35%). In addition, eight expected full siblings (FS) pairs appeared in the PO report with duo error values between 0.55 and 0.91% (data not shown). To inspect these borderline and unknown cases, all pairwise ‘duos’ were classified according to pedigree-based expected relationships (coefficient r>0.25) given by the available pedigree information (**Supplementary Table 3**): PO, FS, grandparents (GP), half siblings (HS), and uncles and aunts (UN). Unknown or more complex relationships were classified as “other” category. A PCA was performed using the variables duo_errors_perc, het_mismatch_A1A2, het_mismatch_A2A1, and perc_mismatches. When all classes were included in the analysis, the large variance of the “other” group dominated the first principal components, obscuring the separation between the other classes. To avoid distortion of the principal component space by highly distant or heterogeneous pairs, the class “other” was therefore included as supplementary individuals (ind.sup) in the PCA. In this way, the main axes were computed only from the primary relationship classes (PO, FS, HS, GP, UN), while “other” pairs were subsequently projected onto the same coordinate system without influencing the eigen decomposition (**Supplementary Figure 1**). This approach maximized the resolution among closely related classes while preserving the spatial representation of more distant or unrelated pairs within the same ordination space. PC2 explained 23.1% of the variance, driven mainly by duo_error_perc (>85%), and clearly separated PO relationships from other classes, including FS pairs misclassified as PO. PC1 accounted for 64.9% of total variance, with the predominant contribution of the heterozygous mismatches variables (>95%), separating FS from the other classes, although partial overlap with HS and GP remained. Within the “other” relationship class, genotypes suspected of misclassification in the genetic diversity analysis were further examined. The two *V. sylvestris* Coste del Cese 1 and *V. sylvestris* Pinnia 2, which clustered with *Vitis* wild species (**Figure 3**), here resulted strictly related with ‘Hutchinson’ and ‘Kober 5 BB’, (data not shown), falling within the PO or the FS confidence regions, respectively.

All expected PPO ‘trios’ (presence of both parents in the panel) were validated, with the exception of the one having IV 8/1 as the offspring (**Supplementary Table 3**). The maximum trio error calculated in this study was 0.91% maintaining the SNPs with Mendelian error but was reduced to 0.59% after their removal (data not shown).

Six chloroplastic SNPs were used to define haplotypes and assess maternal lineage (**Supplementary Table 3**). Three haplotypes (Types 1–3) were identified, with one SNP (AX-691501543) monomorphic (**Supplementary Table 8**). Haplotypes showed near-complete concordance with *V*IVC chlorotypes: among 34 genotypes classified following Arroyo-García et al. (2006), 33 matched chlorotypes A, C, and D with Types 1, 3, and 2, respectively. The only discrepancy involved ‘Riparia grand glabre’, whose chlorotype B could not be resolved with this SNP set and grouped within Type 1. All four rootstocks previously classified according to Riaz et al. (2019) were assigned to chlorotype A.

Integration of SNP-based and *V*IVC chlorotypes allowed maternal lineage to be confirmed for 44 genotypes, remained inconclusive for 37 due to identical parental chlorotypes, and could not be assessed for 61 because parental chlorotypes were unavailable. Parent 1 was excluded as the maternal donor for ‘Terlaner’ and ‘Trebbianina’.

Haplotype distribution in the genetic diversity PCA (**Supplementary Figure 2**) indicated that Type 1 predominated in *V. vinifera* ssp. *sylvestris* and wild *Vitis*, with six *sylvestris* accessions carrying Type 2. In contrast, *V. vinifera* ssp. *sativa* displayed all three chlorotypes.

### 3.4 Validation of candidate polymorphisms for specific traits

Probes were successfully designed for 285 out of the 289 polymorphisms putatively associated with specific phenotypic traits (**Supplementary Table 1A**). To assess their predictive potential, selected SNPs were validated in the BPP and FSP panels, as reported hereafter.

#### 3.4.1 Flower sex

**Figure 4:**
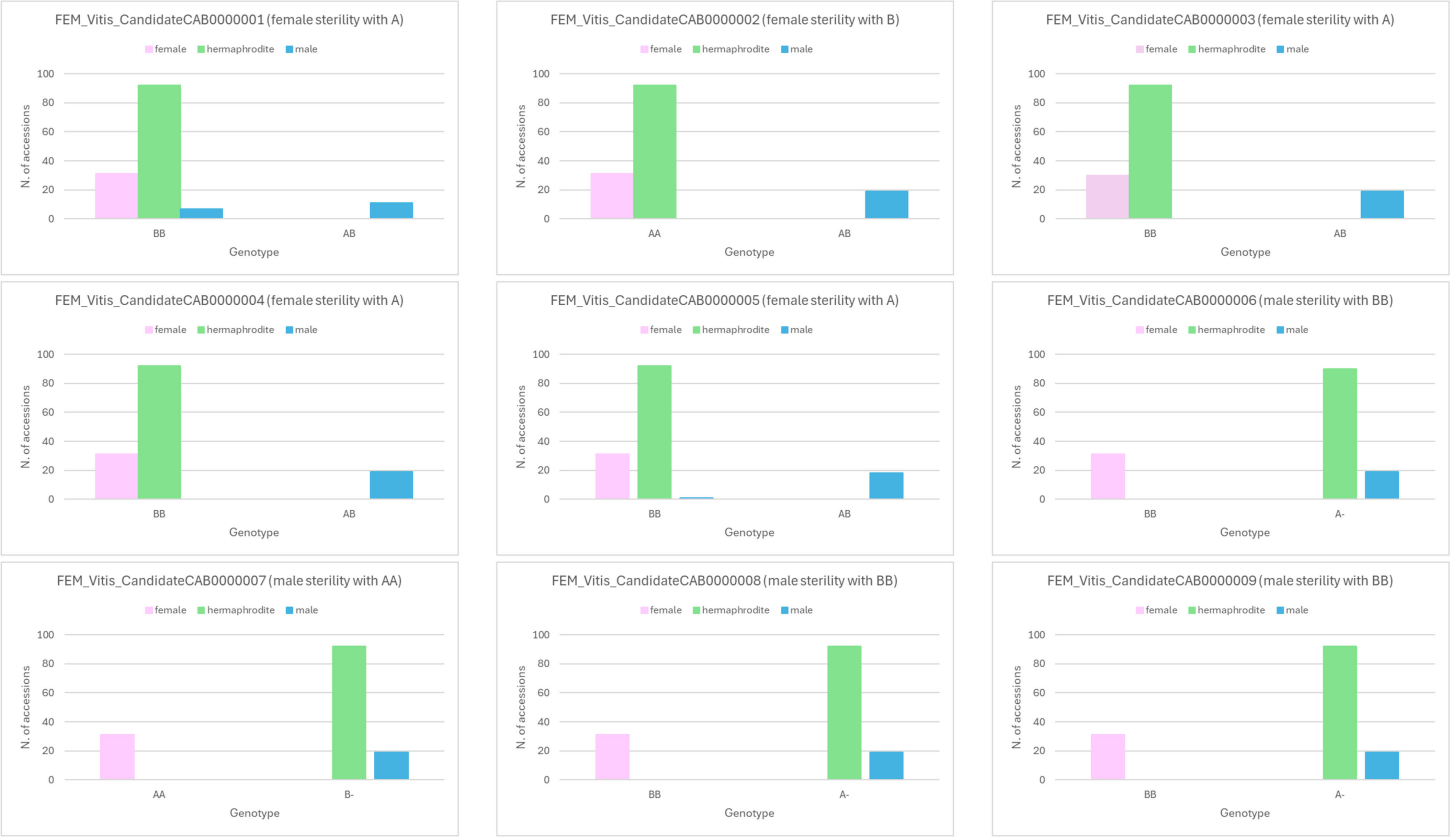
Validation of 9 SNPs associated with flower sex in the breeding parental and flower sex panels without identical genotypes (N = 142).

Seven out of 9 SNPs associated with flower sex (Massonnet et al., 2020) proved to be fully predictive of female sterility (FEM_Vitis_CandidateCAB0000002, FEM_Vitis_CandidateCAB0000003 and FEM_Vitis_CandidateCAB0000004) or male sterility (SNPs from FEM_Vitis_CandidateCAB0000006 to FEM_Vitis_CandidateCAB0000009). Conversely, when employing the SNPs FEM_Vitis_CandidateCAB0000005 and FEM_Vitis_CandidateCAB0000001, flower sex was wrongly predicted in 1 and 7 cases, respectively (**Figure 4**, **Supplementary Tables 3**, **6**). In 12 BPP varieties for which hermaphrodite haplotype (H1f, H2f, H1H1, H1H2) were known (Zou et al., 2021), the haplotype at the 9 SNPs associated with flower sex was compared, resulting in the identification of two SNPs (FEM_Vitis_CandidateCAB0000007 and FEM_Vitis_CandidateCAB0000008) that might determine the Hf or HH asset (**Supplementary Table 9**).

#### 3.4.2 Seed development

**Figure 5:**
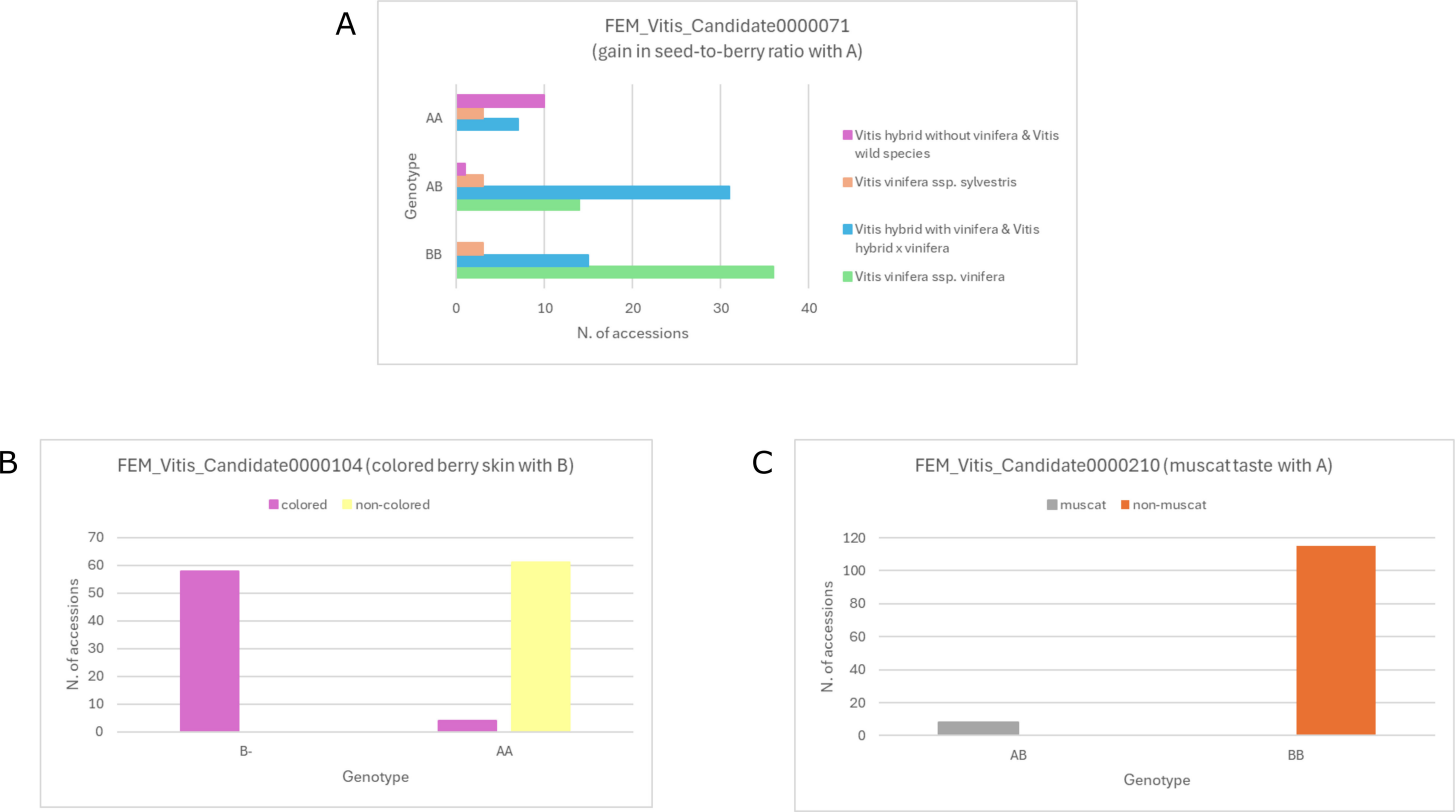
Validation of polymorphisms associated with seed-to-berry ratio **(A)**, berry color **(B)** and muscat taste **(C)** in the non-identical fruit-bearing genotypes of the breeding parental and flower sex panels (N = 123). For **(A)** the genetic background was retrieved from **Supplementary Table 3**. When available, the reclassification based on PCA was employed.

The SNP associated with seed abortion (FEM_Vitis_Candidate0000089) was able to predict the lack of fully developed seeds in ‘Kishmish Vatkana’ and ‘Mars’, while it was not associated with the seedlessness phenotype of ‘Sangiovese seedless’ and ‘Chasselas sans pepins’ (**Supplementary Tables 3**, **6**). The SNP (FEM_Vitis_Candidate0000071) associated with lower seed-to-berry ratios in cultivated varieties compared to wild ones (Magris et al., 2021) was retrieved in all *V. vinifera* ssp. *sativa* genotypes in either homozygous (72%) or heterozygous (28%) condition. The same B allele was also present in the majority (86.5%) of the *Vitis* hybrids with *V. vinifera* ssp. *sativa* in their genetic background and of the *Vitis* hybrid × *V. vinifera* ssp. *sativa* progenies. Conversely, the *Vitis* hybrids without *V. vinifera* ssp. *sativa* in their genetic background and non-*vinifera* showed the wild-type allele (A) in the homozygous state, with only one exception that is Corella 2 (AB). *V. vinifera* ssp. *sylvestris* genotypes displayed quite uniform content of A and B alleles (**Figure 5A**).

#### 3.4.3 Berry color

Despite being classified as “Other” by the Axiom Analysis Suite, one of the three polymorphisms associated with anthocyanin content in berry skin (FEM_Vitis_Candidate0000104, derived from Walker et al., 2007) was found to be a reliable predictor of berry skin coloration (**Figure 5B**; **Supplementary Tables 3**, **6**). All the genotypes with blue black (OIV 225: 6) or dark red violet (OIV 225: 5) berry skin showed at least one B allele (corresponding to a CA sequence in the gene *VvMYBA2*), which was instead absent in the genotypes with green-yellow (OIV 225: 1) or rose (OIV 225: 2) berry skin.

#### 3.4.4 Berry taste

The SNP associated with berry muscat taste (FEM_Vitis_Candidate0000210, derived from Emanuelli et al., 2010) was present in all the 8 muscat genotypes (**Figure 5C**; **Supplementary Tables 3**, **6**), while, as expected, it was not linked to the specific aroma of ‘Chardonnay Blanc Musque’, ‘Gewuerztraminer’ and ‘Calardis Musque’ (muscat-like aromatic). The unique mutations of ‘Chardonnay Blanc Musque’ (FEM_Vitis_Candidate0000211) and ‘Gewuerztraminer’ (FEM_Vitis_Candidate0000213) were also confirmed (**Supplementary Table 6**), differentiating these aromatic genotypes from their neutral clones ‘Chardonnay Blanc’ and ‘Savagnin Rose/Blanc’ (data not shown). The unique mutation of ‘Chardonnay Blanc Musque’ was inherited by one out of four ‘Chardonnay Blanc Musque’ offspring present in the BPP (GIA F12P140), which was however found to have a neutral taste. Conversely, the unique mutation of ‘Gewuerztraminer’ was not retrieved in its offspring ‘Petra’ and F49P35, in line with their non-aromatic taste. This mutation was instead present in a number of other genotypes (Coia 05, ‘Bronner’, Coia 01, Coia 09, Corella 2, ‘Felicia’, GIR F2P97, INF 12P128, ‘Mars’, ‘Merzling’, ‘Nermantis’, ‘Seyval Blanc’, ‘Solaris’, ‘Kober 5BB’, ‘Riparia Gloire de Montpellier’, ‘Rupestris du Lot’, ‘Rupestris metallique’, *V. sylvestris* Pinnia 2), some of which with male flowers (**Supplementary Table 6**). Out of 15 SNPs suggested as molecular markers in breeding programs to obtain varieties with appropriate levels of methoxypyrazines (Guillaumie et al., 2013), four were discarded after analysis with the Axiom Analysis Suite. All the remaining ones were confirmed in the so-called Bordeaux cultivars that are particularly rich in methoxypyrazines (‘Cabernet Franc’, ‘Cabernet Sauvignon’, ‘Carménère’, ‘Merlot Noir’, and ‘Sauvignon Blanc’) and were found to belong to two possible haplotypes (**Supplementary Table 6**). One haplotype is shared by ‘Cabernet Franc’ and ‘Cabernet Sauvignon’, the other one by ‘Carménère’, ‘Merlot Noir’, and ‘Sauvignon Blanc’. The latter haplotype was also found in the following additional genotypes: ‘VRH3082-1-42’ (with ‘Merlot Noir’ in its pedigree), ‘Cabernet Cortis’ (with ‘Cabernet Sauvignon’ as parent), GIR F2P97 (with VRH3082-1–42 in its pedigree), ‘Lambrusco di Sorbara’, ‘Picolit’ and five, *V. vinifera* ssp. *sylvestris*, three of which with male flowers (**Supplementary Table 6**).

#### 3.4.5 Bunch compactness

A heterozygous mutation (FEM_Vitis_Candidate0000065) specific to ‘Pinot Noir’ loosely clustered clones (Rossmann et al., 2020) was exclusively found in the genotype ‘Civit 2’, which has ‘Pinot Noir’ as a parent and displays a loose cluster (**Supplementary Table 6**).

### 3.5 Analysis with previous markers

The analysis of the previously available markers linked to flower sex (VVIB23) and berry color (VvmybA1) loci is reported in **Supplementary Table 3**. As regards flower sex, the allele 284 was found in 80 out of 94 genotypes with hermaphrodite flower type, while the alleles 304, 306 or 308 were detected in 17 out of 19 genotypes with male flower type, with a dominant effect and a prevalence of the allele 304. Only the presence of the allele 290 in homozygosity seems to be clearly predictive of female flower type. With respect to berry color, 64 out of 66 genotypes with codes 1 and 2 for OIV descriptor 225 were correctly predicted as having non-colored berries based on the presence of the VvmybA1^ITA^ allele in homozygosity. The only exceptions were ‘Chaouch Rozovyi’ and ‘Moscatel Rosado’, which clearly displayed also the VvmybA1^ALF^ allele (data not shown). Out of the 59 genotypes with codes 5 and 6 for OIV descriptor 225, 45 were correctly predicted as having colored berries based on the presence of the VvmybA1^ALF^ allele in heterozygosity or homozygosity. Fourteen additional genotypes were classified as colored according to the presence of putative functional alleles different from VvmybA1^ALF^ (marked with § in **Supplementary Table 3**).

## 4 Discussion

### 4.1 Novelty of the Axiom^®^Vitis22K SNP array

Genotyping arrays are powerful tools to explore genetic diversity, relatedness, and clustering among grapevine cultivars, as well as to investigate the genetic architecture of important phenotypic traits and enable targeted breeding efforts. Several SNP arrays have been developed for many crops over the years, including maize (Unterseer et al., 2014), apple (Bianco et al., 2016), pear (Montanari et al., 2019), walnut (Marrano et al., 2019), and citrus (Hiraoka et al., 2024). Recently, a high-density 200K Axiom SNP array was developed using resequencing data from 313 *Vitis* genotypes and one *Ampelopsis* genotype (Zhang et al., 2025), and was shown to support high-resolution genetic mapping and marker-trait association analyses. However, such high-density platforms are primarily optimized for fine-scale genomic analyses and may be less suited for routine genotyping, comparative studies, and molecular breeding programs, particularly when cost, sample management flexibility, and compatibility with existing datasets are critical considerations. In this context, the Axiom^®^Vitis22K array described here was specifically designed as a complementary and application-oriented platform. By leveraging a genetically diverse discovery panel, the array ensures broad applicability across a wide range of genetic backgrounds, including multiple grapevine species, while providing genome-wide coverage with a substantial yet manageable number of markers (**Figure 2**). A key advantage of the Vitis22K array is the inclusion of 11,701 SNPs derived from the GrapeReSeq 18K Vitis chip, which was extensively used in previous studies. Although the latter is no longer commercially available, incorporating these markers ensures continuity with historical datasets and preserves the value of previously generated genotypic information, thereby facilitating data integration across studies and over time. In addition, compared with previous platforms, the new array also contains candidate SNPs associated with target traits and offers enhanced operational flexibility through its inclusion in a 70K multispecies array (grapevine, chestnut, hazelnut and black walnut; Bianco et al., 2023). This multispecies configuration allows samples from different species to be simultaneously processed on the same plate, simplifying laboratory logistics and enabling cost-effective genotyping of small populations that would otherwise not justify the use of a full 96-well plate.

### 4.2 Evaluation of SNP call quality and cross-platform concordance

Among the 21,874 variants included in this Axiom^®^Vitis22K array between the 67 and 68% for all the three sets of SNPs (SNPs associated with traits of interest, resequencing-derived SNPs, and GrapeReSeq 18K Vitis chip-derived SNPs) were classified as PolyHighResolution, MonoHighResolution and NoMinorHom (i.e. the ‘recommended’ classes) by the Axiom Analysis Suite. Variants were well scattered across the genome, enabling dense coverage information for downstream investigations such as haplotype reconstruction in related individuals when integrated with sequencing data, as well as pedigree reconstruction.

Although 499 variants were initially classified as not recommended by the ThermoFisher scoring procedure (i.e. variants with probes difficult to design or containing additional SNPs), 85 were recovered as PASS by AxioSAFE, most of which originated from the GrapeReSeq 18K Vitis chip and had been identified in wild species.

Concordance analysis between the Illumina Infinium and Thermo-Fisher Axiom platforms based on 27 genotypes shared samples confirmed high reproducibility between technologies. Specifically, the mean concordance rate of 97.91% in the case of the new Axiom^®^Vitis22K and the Infinium GrapeReSeq18K, slightly exceeded the 97.1% previously reported in apple (Howard et al., 2021).

### 4.3 Insights from genetic diversity and kinships

Two genotype pairs were identified as identical. The Kozma 01-1–9 and Kozma 01-1–93 accessions were wrongly included in the BPP as siblings, while ‘Sangiovese’ and ‘Sangiovese seedless’ are two somatic variants (Nwafor et al., 2014; Costantini et al., 2021). The difficulty in distinguishing bud sports using array-based genotyping is expected, as somatic mutations occur at a much lower frequency than the variation observed among cultivars and may also be restricted to specific meristematic cell layers (Urra et al., 2023; Callipo et al., 2025).

The relationships identified by AxioSAFE were largely consistent with known pedigrees, while highlighting two mistakes in the reported pedigrees of ‘Petra’ and of IV 8/1. In the case of ‘Petra’, SSR markers and SNP-based analysis indicated ‘Gewuerztraminer’ instead of ‘Pinot Noir’ (from *V*IVC) as the true parents, in agreement with direct communication from the breeder (Pál Kozma). The PO analysis also provided novel hypotheses for unresolved pedigrees. One notable case involves ‘Savagnin Blanc’ and its somatic variant ‘Gewuerztraminer’. ‘Savagnin Blanc’ is recognized as the male progenitor of ‘Pinot’ (Ramos-Madrigal et al., 2019; Magris et al., 2021), and the pedigree of ‘Manzoni Bianco’ is confirmed by SSR markers (‘Riesling Weiss’ × ‘Pinot Blanc’). The observation that the ‘Savagnin Blanc’ somatic variant ‘Gewuerztraminer’ showed only 0.35% Mendelian error with ‘Manzoni Bianco’ suggests that the apparent excess contribution from the grandparent ‘Savagnin Blanc’ to ‘Manzoni Bianco’ may result from the presence of ‘Savagnin Blanc’ or a closely related genotype on the ‘Riesling Weiss’ side of the ‘Manzoni Bianco’ pedigree i.e., as a grandparent of ‘Heunisch Weiss’ and/or of its maternal unknown parent. Another interesting case in which the PO was useful to investigate the genetic background of the accessions regards the two *V. sylvestris* Coste del Cese 1 and *V. sylvestris* Pinnia 2 (**Supplementary Figure 3**), clustering with non-*vinifera Vitis* instead of *V. vinifera* ssp. *sylvestris* in the genetic diversity PCA. This is surprising since those accessions derived from a field campaign taken in Italy. Here the PO analysis revealed the two accessions having a very close relationship with ‘Kober 5 BB’ and ‘Hutchinson’, respectively, suggesting the hypothesis that those are probably abandoned rootstocks or dispersed seed of rootstocks open pollination. A similar scenario applies to *V. sylvestris* Lauri 1, which clusters with the ssp. *sativa*, and whose cluster morphology is more similar to that of *V. vinifera* ssp. *sativa* than to that of *sylvestris* (**Supplementary Figure 4**) and likely represents a seed dispersed by open pollination from a local *sativa* variety. This hypothesis will be further investigated by including *V. sylvestris* Lauri 1 in a future, more extensive genetic study. Two more genotypes resulted in misclassification in the genetic diversity PCA. Finally, the close genetic affinity of ‘Lambrusco di Sorbara’ with *V. vinifera* ssp. *sylvestris* is consistent with previous findings showing that several Lambrusco varieties carry an introgressed *sylvestris* component (Schneider et al., 2024), and in particular ‘Lambrusco di Sorbara’ is linked with *sylvestris* and *sativa* lineages from Western Europe (Magris et al., 2021).

Although chlorotypes exhibit limited variability in grapevine (Arroyo-García et al., 2006), they are useful to resolve maternal lineages, since the plastids are maternally inherited. In this work, six chloroplastic SNPs were used for this purpose, detecting three different chloroplast haplotypes. Although it was not suitable to distinguish between chlorotype A and B, they were useful to verify maternal lineages. In case of diverse chlorotype between the parents, all crossbreeding directions were confirmed, while for ‘Terlaner’ and ‘Trebbianina’ the known parent was excluded as possible mother.

### 4.4 Validation of polymorphisms for specific traits

The Axiom^®^Vitis22K SNP array incorporates several candidate markers that enable trait-oriented genotyping. Predictive potential was demonstrated for flower sex, seed development, berry color and taste.

Seven of nine SNPs in the sex locus (Massonnet et al., 2020) were found to be fully predictive of flower sex in the panels under study (**Figure 4**). Out of two partially predictive SNPs, one (FEM_Vitis_CandidateCAB0000005) was able to predict female sterility in all male genotypes, with the only exception of ‘Cinerea Arnold’, which suggests the existence of a specific mutation in this genotype and warrants further investigation. The second SNP (FEM_Vitis_CandidateCAB0000001), when analyzed in a wider dataset, i.e. the whole FEM germplasm collection, was found instead to correctly predict all male genotypes included in the present study (data not shown). This indicates that the predictive value of this SNP depends on the analyzed panel. Moreover, the position of the clusters in the Signal plot of the Axiom Analysis Suite suggests the existence of null alleles or a paralogous locus, whose investigation is out of scope of this study. The availability of at least seven SNPs predictive of flower sex represent a major step forward compared to markers available so far. Indeed, even if specific size variants of the VVIB23 microsatellite were previously found to be associated with the sex alleles H (284), f (288 and 290, the latter in the homozygous state) and M (304 and 308), a number of exceptions were observed in those works and here, which are due to the occurrence of recombination between the sex locus and VVIB23 locus, as well as to the chance of allele size homoplasy (Battilana et al., 2013; Perko et al., 2024). In the present study, this is especially true for the allele 288, which did not help to predict the genotypes with female flower type. Moreover, the investigation of wild grapevines highlighted the existence of several VVIB23 alleles, for a number of which a statistically significant association with flower sex has not been established (Belkina et al., 2025). To overcome all these limits, a combination of different genetic markers for sex determination was necessarily adopted in some studies (Riaz et al., 2018; Perko et al., 2024). Out of the above seven SNPs predictive of flower sex, two might also discriminate the Hf or HH asset, unlike the microsatellite VVIB23. The ability to recognize hermaphrodite plants with a female sex allele is relevant in grapevine breeding programs, given that up to 50% of ff × Hf progeny populations have individuals with female flowers which are of no commercial use, since they require a male or hermaphrodite vine nearby to provide pollen to set the fruit (Battilana et al., 2013).

The SNP associated with seed abortion (FEM_Vitis_Candidate0000089) was confirmed to predict the lack of fully developed seeds in ‘Kishmish Vatkana’ and ‘Mars’. Its inability to predict the seedlessness phenotype of ‘Sangiovese seedless’ and ‘Chasselas sans pepins’ is not surprising, as this SNP was reported to cause stenospermocarpy within ‘Sultanina’ genetic background (Royo et al., 2018), whereas different seedlessness sources were hypothesized for ‘Corinto Nero’ (‘Sangiovese seedless’) parthenocarpy and for ‘Chasselas apyrène’ (‘Chasselas sans pepins’) stenospermocarpy (Costantini et al., 2021). The genotype at the SNP (FEM_Vitis_Candidate0000071) associated with lower seed-to-berry ratios in cultivated varieties compared to wild ones (Magris et al., 2021) was largely consistent with the expectations based on the genetic background of the genotypes under study (**Figure 5A**). The only exception of Corella 2 (classified as non-*vinifera* but showing the B allele) might be explained by a wrong classification. Corella 2 is indeed the closest genotype to the *Vitis* hybrids with *V. vinifera* ssp. *sativa* in their genetic background, therefore we cannot exclude that it is a hybrid with *V. vinifera* ssp. *sativa* itself. Among the 6 V*. vinifera* ssp. *sylvestris* genotypes with at least one B allele, three (*V. sylvestris* cl. Guemuld 103-64, *V. sylvestris* cl. Guemuld 105–6 and 5c (2004)) are located in admixed space encompassing both *V. vinifera* ssp. *sativa* and *V. vinifera* ssp. *sylvestris* in the PCA, while the other three clearly belong to *V. vinifera* ssp. *sylvestris* but show well-developed berries with a discrete size (**Supplementary Figure 5**).

The marker FEM_Vitis_Candidate0000104 proved to reliably predict berry color in widely different genetic backgrounds (**Figure 5B**), which is far more convenient compared to the alternative system based on sequence variation at the *VvMYBA1* locus. Indeed, the latter system must take into account the existence of different functional alleles in different genotypes (Lijavetzky et al., 2006), as occurring also in the present study. The most likely explanation for varieties with rose berry skin (‘Gewuerztraminer’, ‘Souvignier Gris’, ‘Chaouch Rozovyi’ and ‘Moscatel rosado’) bearing the same FEM_Vitis_Candidate0000104 allele as the non-colored genotypes is that they gained berry skin color through a distinct mechanism.

The SNP associated with berry muscat taste (FEM_Vitis_Candidate0000210, **Figure 5C**), and the unique mutations of ‘Chardonnay Blanc Musque’ (FEM_Vitis_Candidate0000211) and ‘Gewuerztraminer’ (FEM_Vitis_Candidate0000213) were also confirmed. The unique mutation of ‘Chardonnay Blanc Musque’ was also found to be heritable, but the genotype displaying the SNP (GIA F12P140) was non-aromatic when tasted. This finding as well as the presence of the unique mutation of ‘Gewuerztraminer’ in a number of non*-vinifera* genotypes, some of which with male flowers, likely indicate that the two unique mutations of ‘Chardonnay Blanc Musque’ and ‘Gewuerztraminer’ are not predictive of an aromatic taste out of the *V. vinifera* ssp. *sativa* taxon or the muscat-like taste is masked by the complex aromatic background of hybrids and wild species. A decisive answer could be provided only by a metabolomic analysis, which is planned on the panels under study. Similarly, the haplotype of ‘Carménère’, ‘Merlot Noir’, and ‘Sauvignon Blanc’ at 11 SNPs associated with methoxypyrazine content was also found in 10 additional genotypes. However, in the absence of further chemical data no conclusion can be drawn on the predictive value of these markers.

Finally, the SNP FEM_Vitis_Candidate0000065 proved to be able to predict loose cluster architecture in ‘Pinot Noir’ clones and their offspring, like Civit 2 (**Supplementary Figure 6**). The availability of this marker may be convenient in clonal selection and breeding programs, as loose cluster bunches are more resilient to bunch disease, for example *Botrytis cinerea* infections, and tend to have a uniform berry ripening (Rossmann et al., 2020).

## 5 Conclusion

In this study, we developed a new Axiom^®^ SNP array, comprising 22K markers evenly distributed over the 19 grapevine chromosomes. The array was designed through a stringent SNP selection strategy to maximize polymorphism and technical robustness, ensure representation of *Vitis* genetic diversity, and include SNPs with demonstrated or potentially predictive value for different target traits (i.e. abiotic and biotic stresses, agronomical, biochemical, phenological, and morphological). This tool will greatly benefit grapevine molecular breeding especially when involving the analysis of interspecific hybrids, naturalized or derived from crossbreeding programs. Importantly, the integration of SNPs from the discontinued Illumina GrapeReSEq 18K chip ensures backward compatibility with historical datasets.

When tested on two dedicated panels, the Axiom^®^Vitis22K SNP array showed high concordance with the Illumina array and efficiently captured genetic diversity, resolved pedigree relationships, and predicted target phenotypes. These results confirm its suitability for a wide range of genetics and genomics applications.

Overall, the developed array provides a novel tool that supports the characterization and management of grapevine germplasm, facilitates high-resolution linkage mapping, enhances the dissection of the genetic architecture of complex traits, and strengthens marker-assisted breeding strategies to improve the quality and sustainability of grapevine cultivars.

## Data Availability

The datasets presented in this study can be found in **Supplementary Material**.

## Footnotes

1
https://www.vivc.de

2
https://urgi.versailles.inra.fr/Species/Vitis/Genome-Browser

3
https://www.grapegenomics.com/pages/VvCabSauv/jbrowse2.php

4
https://forge.inrae.fr/urgi/is/ontologies/-/tree/develop/Vitis?ref_type=heads

5
https://www.oiv.int/what-we-do/amp/225

## Supplementary material

Supplementary Figure 1 Principal Component Analysis (PCA) of pairwise genetic relatedness showing the distribution of relationship categories. The first two principal components (PC1 and PC2) explain 64.9% and 23.1% of the total variance, respectively. Points represent pairwise comparisons classified as parent-offspring (PO), full-sibling (FS), half-sibling (HS), grandparent-grandchild (GP), or unrelated (UN). Colored ellipses indicate 95% confidence intervals for each relationship group. The ordination reveals clear separation between close relationships (PO, FS) and more distant or unrelated pairs (HS, GP, UN).

Supplementary Figure 2 Principal Component Analysis (PCA) based on SNP data illustrating the genetic diversity of the studied panel with colors indicating the chlorotypes. The clustering pattern of chlorotypes compared with the pattern of the taxonomic classes (**Figure 2**) highlighted *V. vinifera* ssp. *sylvestris* and wild American *Vitis* carrying predominantly Type 1, while *V. vinifera* ssp. *sativa* carrying all three types.

Supplementary Figure 3 Leaves of two *V. vinifera* ssp. *sylvestris* genotypes (*V. sylvestris* Coste del Cese 1 and *V. sylvestris* Pinnia 2) clustering with wild *Vitis*.

Supplementary Figure 4 Leaves and cluster of one *V. vinifera* ssp. *sylvestris* genotype (*V. sylvestris* Lauri 1) clustering with the group of *V. vinifera* ssp. *sativa.*

Supplementary Figure 5 Clusters of *V. vinifera* ssp. *sylvestris* genotypes (*V. sylvestris* Coste Latrive 2, *V. sylvestris* Peronello and *V. sylvestris* Pinocchio 1) showing at least one B allele at SNP FEM_Vitis_Candidate0000071.

Supplementary Figure 6 Loose clusters of ‘Civit 2’.

Supplementary Table 1 A) Polymorphisms selected for inclusion in the new Axiom^®^Vitis22K SNP array based on their putative association with relevant phenotypic traits. For each polymorphism are reported the corresponding probeset ID, the position on the + strand of the reference genome assembly PN40024 12X.v2 or on the + strand of the CabSauv08 v.1.1 phased diploid genome assembly, the published polymorphism name and type, the published affected gene and the corresponding strand, the type of variant tissue, the effect of the variant allele (as reported in columns D and E) on the phenotype (as reported in column O), the affected phenotypic variable as indicated in the paper and as classified according to the Vitis Ontology V2 when feasible (na = not available), the source of variation (the genotype(s) where the polymorphism was originally identified), the reference paper, the polymorphism classification by the Axiom Analysis Suite (AxAS) and by AxioSAFE. In case of multiple affected phenotypic variables (in column O), a unique effect of the variant allele is reported when common among variables, otherwise, different effects are reported separated by commas in the same order as the variables. B) Polymorphisms putatively associated with specific phenotypic variables for which probe design was not successful.

Supplementary Table 2 Summary of whole-genome sequencing resources utilized in this study. The table lists sample identifiers, species classifications, and genotype accession IDs for 12 *Vitis* accessions sequenced using Illumina PE150 or PacBio technologies. Estimated sequencing coverage (X-fold) and filtered paired-end read lengths are reported along with the corresponding reference publications.

Supplementary Table 3 List of genotypes included in the Breeding Parental Panel (BPP) and in the Flower Sex Panel (FSP). For each genotype are reported the internal code, the accession number in FEM germplasm collection, the name (uppercase if corresponding to a *V*IVC prime name, otherwise lowercase), the code of hybridization on the Axiom^®^Vitis22K SNP array, the occurred hybridization on the GrapeReSeq 18K Vitis chip (x), the phenotype related to flower sex, seed development, berry color and taste, the taxon and genetic background, the pedigree as reported at *V*IVC or by FEM breeders (historical pedigree expressed with prime names, when possible) and as computed with SNP data, and the maternal parent (when imputable). Next to flower sex, VVIB23 alleles are reported with predictive sizes in boldface - green for hermaphrodite, pink for female and light blue for male flower types, respectively (Battilana et al., 2013, Perko et al., 2024) - and exceptions in red. Berry color is classified according to the OIV descriptor 225 (1 = green yellow, 2 = rose, 3 = red, 4 = grey, 5 = dark red violet, 6 = blue black) and is also predicted based on *VvMybA1* alleles (Lijavetzky et al., 2006). The presence of putative functional alleles different from VvmybA1ALF is indicated by §. Berry taste is marked by # when it does not coincide with that reported at *V*IVC. The term “Vitis complex hybrid” in the genetic background defines a hybrid with *V. vinifera* ssp. *sativa* and *M. rotundifolia* in the background. The reported pedigree has a different apex when it is confirmed by the universal set of microsatellite markers (see footnote). Abbreviations: F = female, H = hermaphrodite, M = male, MPs = methoxypyrazines, nd = not detected.

Supplementary Table 4 Number of SNP markers selected from the previous GrapeReSeq 18K Vitis genotyping chip and included in the new Axiom^®^Vitis22K SNP array, with the corresponding number of probes.

Supplementary Table 5 Summary of SNP marker information and quality control status. The table includes the probeset identification code (Probeset_ID), SNP name (SNP_ID), chromosome assignment (Chromosome), physical position on the reference genome assembly PN40024.T2T (v5) (Position), SNP source (SNP Origin), source species (Species of origin), indication of whether the SNP was represented by a double probe (Double Probe), Axiom^®^ Analysis Suite conversion type (AxAS ConversionType), and the final status assigned by AxioSAFE (AxioSafe status).

Supplementary Table 6 SNPs from the Axiom^®^Vitis22K SNP array that passed the AxioSAFE filtering applied to the accessions of the Breeding Parental and the Flower Sex panels. The 13 SNPs marked with * did not pass the AxioSAFE but were manually recovered because they belong to the predictive candidate polymorphisms for specific traits discussed in the text. Abbreviation: A0 = major allele, A1 = minor allele, NC= NoCall.

Supplementary Table 7 A) Technology comparison for SNPs genotyped with the GrapeReSeq 18K Vitis genotyping and the new Axiom^®^Vitis22K SNP arrays. The number of matching genotypes, the total genotypes and the percentage of matching genotypes are reported next to the sample identification codes (VV for *V. vinifera* and VH for *Vitis* hybrids) and names. B) Technology comparison for SNPs genotyped with the GrapeReSeq 18K Vitis genotyping and the new Axiom^®^Vitis22K SNP arrays after the SNP filtering with AxioSAFE: only “PASS” and “MONO_HIGH_RESOLUTION” variants are considered. The number of matching genotypes, the total genotypes and the percentage of matching genotypes are reported next to the sample identification codes and names.

Supplementary Table 8 Chloroplastic SNPs and maternal lineage validation. Here are reported the chlorotypes determined in this work (1,2,3) and their translation into the *V*IVC classification of Arroyo-García et al. (2006). When the correspondence is not respected, the translated *V*IVC chlorotype is given in red. Parental lines that were corrected based on the current family relationship study are labeled with *.

Supplementary Table 9 Combination of SNPs associated with flower sex in the twelve varieties, whose hermaphrodite haplotype could be retrieved from Zou et al. (2021). The SNPs FEM_Vitis_CandidateCAB0000007 and FEM_Vitis_CandidateCAB0000008 are candidates to discriminate the Hf or HH asset, unlike the microsatellite VVIB23 (in red is an example of genotype shared by Hf and HH).
