# Supplementary figures and images for: An advanced genotyping tool to inspect grapevine variability: the Axiom®Vitis22K SNP array
Source: Front Plant Sci. 2026 Mar 18;17:1771381. doi: 10.3389/fpls.2026.1771381 (PMC13041636; doi:10.3389/fpls.2026.1771381)

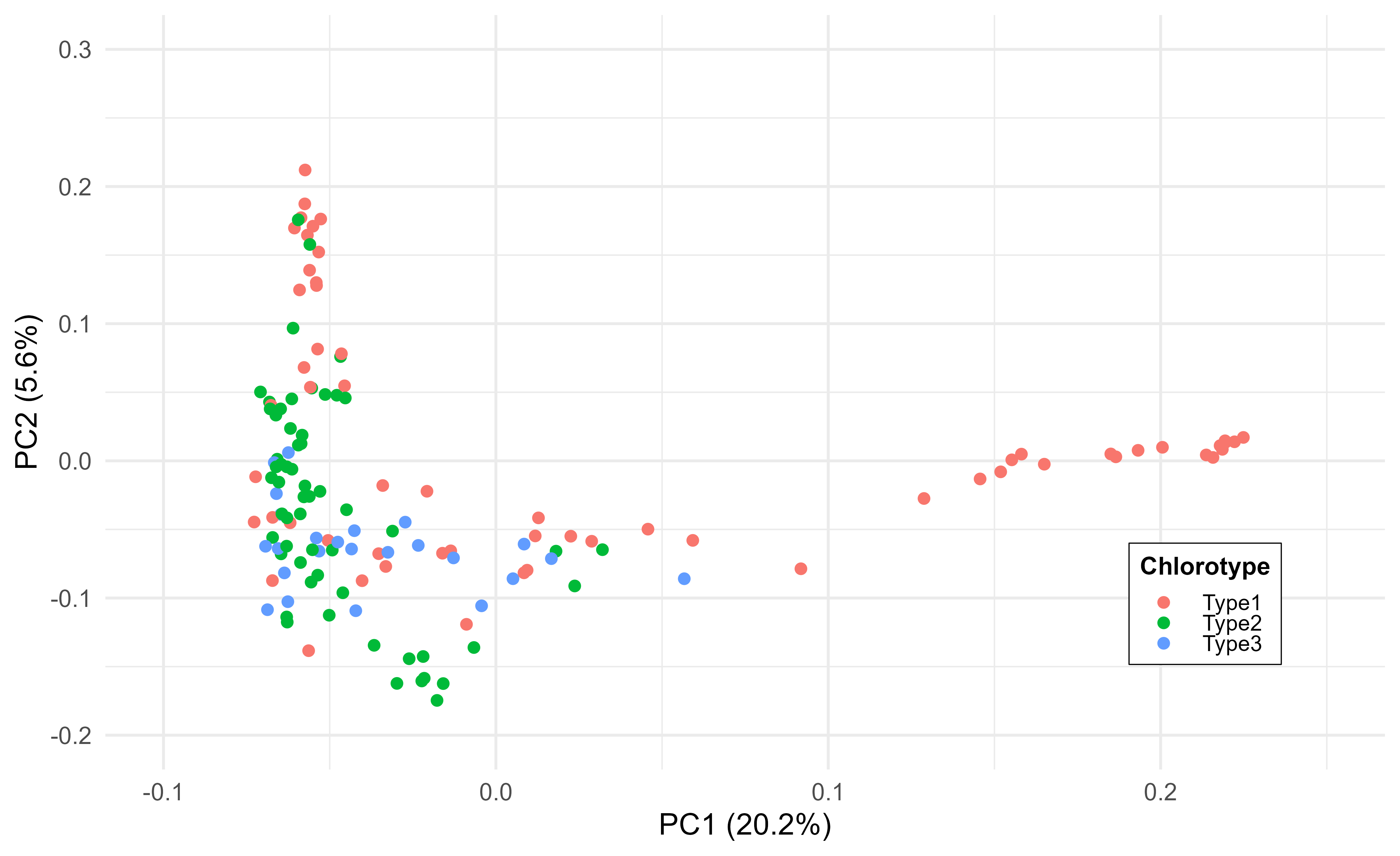

Supplement: Supplementary Figure 1 — Principal Component Analysis (PCA) of pairwise genetic relatedness showing the distribution of relationship categories. The first two principal components (PC1 and PC2) explain 64.9% and 23.1% of the total variance, respectively. Points represent pairwise comparisons classified as parent-offspring (PO), full-sibling (FS), half-sibling (HS), grandparent-grandchild (GP), or unrelated (UN). Colored ellipses indicate 95% confidence intervals for each relationship group. The ordination reveals clear separation between close relationships (PO, FS) and more distant or unrelated pairs (HS, GP, UN). [file DataSheet1.zip › Data Sheet/Sup. Figure 2.png]

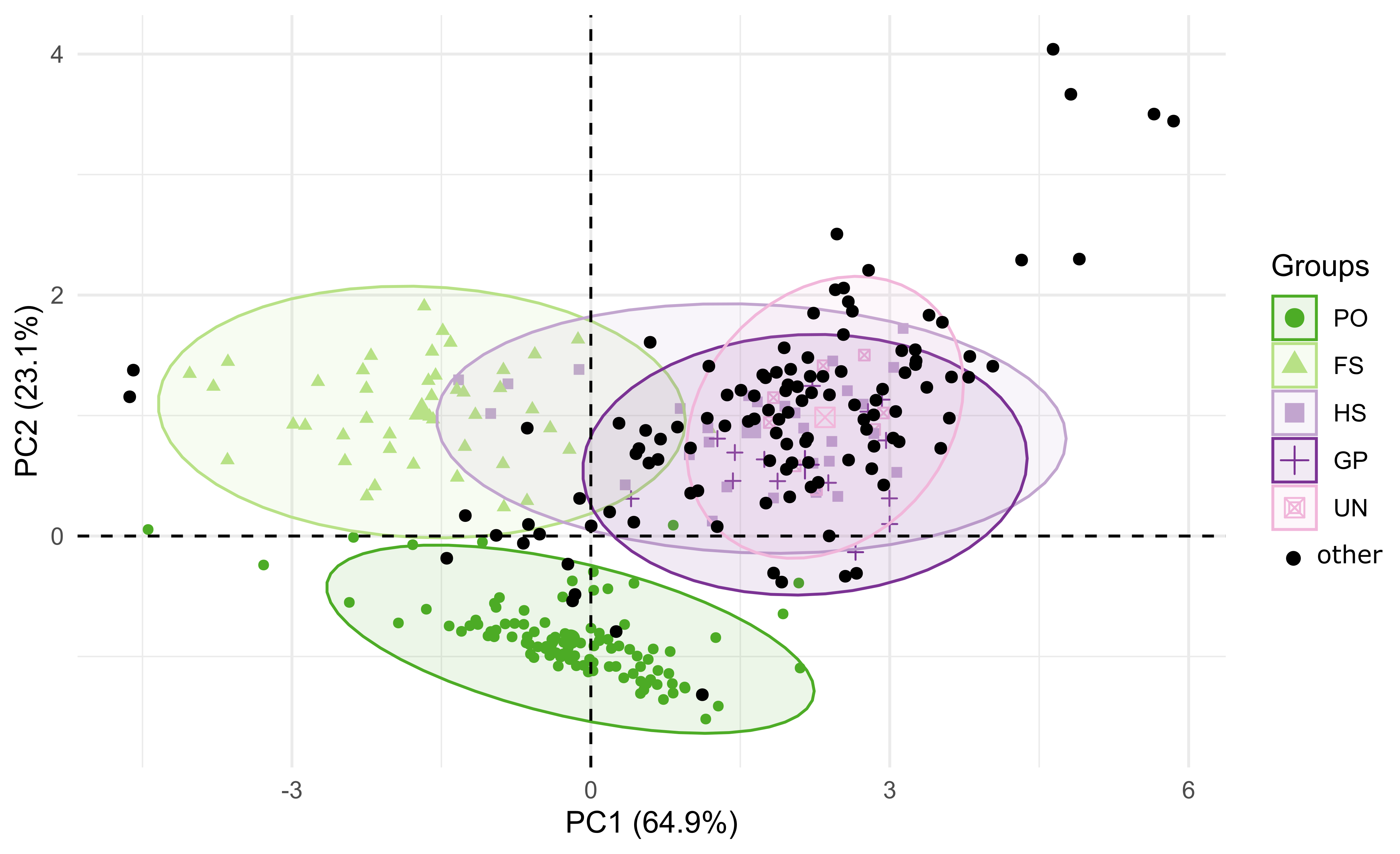

Supplement: Supplementary Figure 1 — Principal Component Analysis (PCA) of pairwise genetic relatedness showing the distribution of relationship categories. The first two principal components (PC1 and PC2) explain 64.9% and 23.1% of the total variance, respectively. Points represent pairwise comparisons classified as parent-offspring (PO), full-sibling (FS), half-sibling (HS), grandparent-grandchild (GP), or unrelated (UN). Colored ellipses indicate 95% confidence intervals for each relationship group. The ordination reveals clear separation between close relationships (PO, FS) and more distant or unrelated pairs (HS, GP, UN). [file DataSheet1.zip › Data Sheet/Sup. Figure 1.png]

## Slide 1
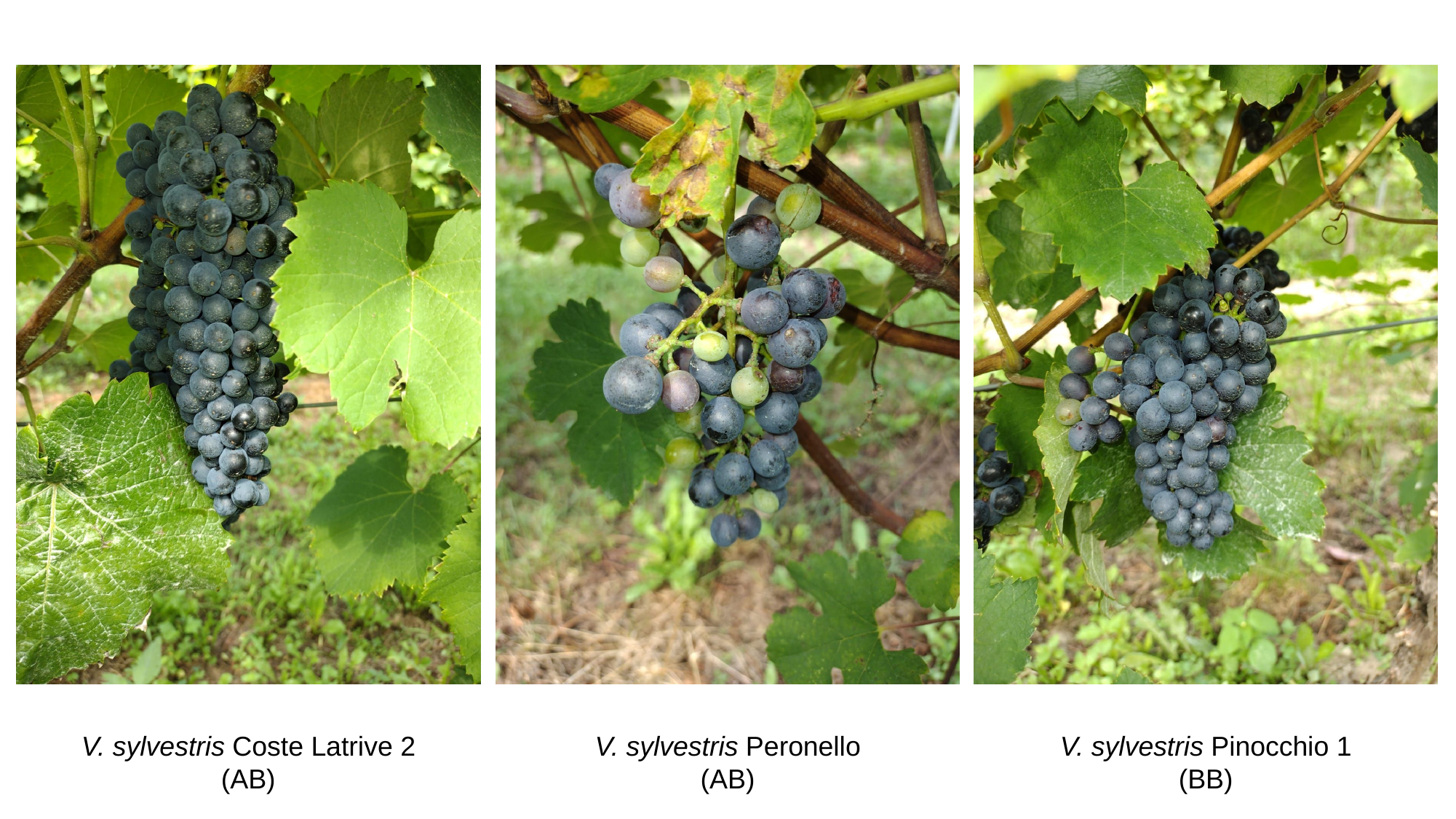

V. sylvestris Coste Latrive 2
(AB)
V. sylvestris Peronello
(AB)
V. sylvestris Pinocchio 1
(BB)

Supplement: Supplementary Figure 1 — Principal Component Analysis (PCA) of pairwise genetic relatedness showing the distribution of relationship categories. The first two principal components (PC1 and PC2) explain 64.9% and 23.1% of the total variance, respectively. Points represent pairwise comparisons classified as parent-offspring (PO), full-sibling (FS), half-sibling (HS), grandparent-grandchild (GP), or unrelated (UN). Colored ellipses indicate 95% confidence intervals for each relationship group. The ordination reveals clear separation between close relationships (PO, FS) and more distant or unrelated pairs (HS, GP, UN). [file DataSheet1.zip › Data Sheet/Sup. Figure 5.pptx]

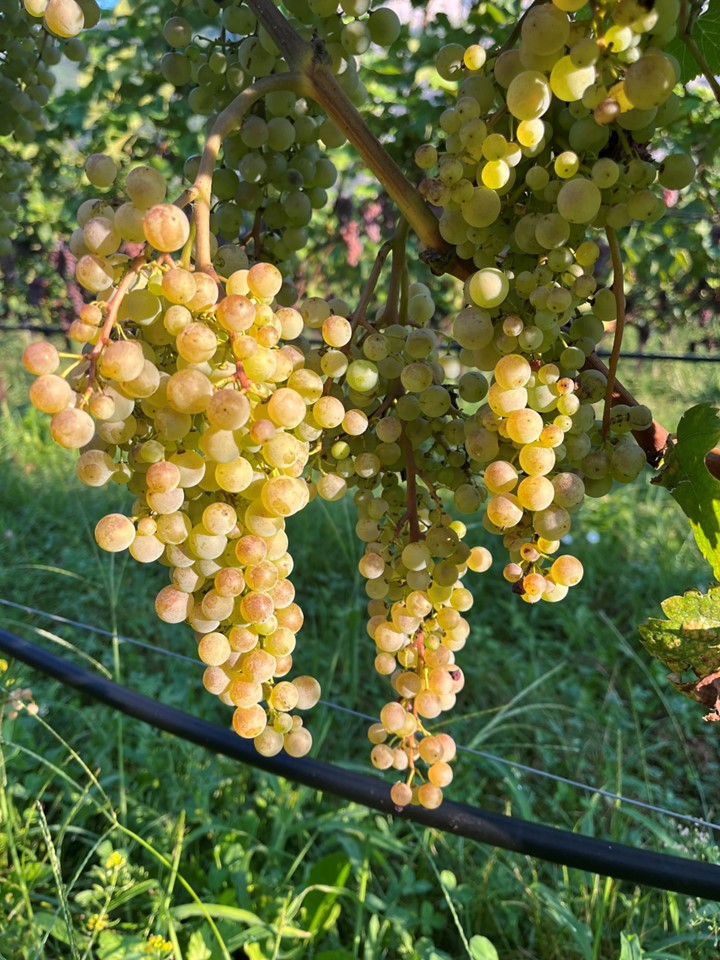

Supplement: Supplementary Figure 1 — Principal Component Analysis (PCA) of pairwise genetic relatedness showing the distribution of relationship categories. The first two principal components (PC1 and PC2) explain 64.9% and 23.1% of the total variance, respectively. Points represent pairwise comparisons classified as parent-offspring (PO), full-sibling (FS), half-sibling (HS), grandparent-grandchild (GP), or unrelated (UN). Colored ellipses indicate 95% confidence intervals for each relationship group. The ordination reveals clear separation between close relationships (PO, FS) and more distant or unrelated pairs (HS, GP, UN). [file DataSheet1.zip › Data Sheet/Sup. Figure 6.jpg]

## Slide 1
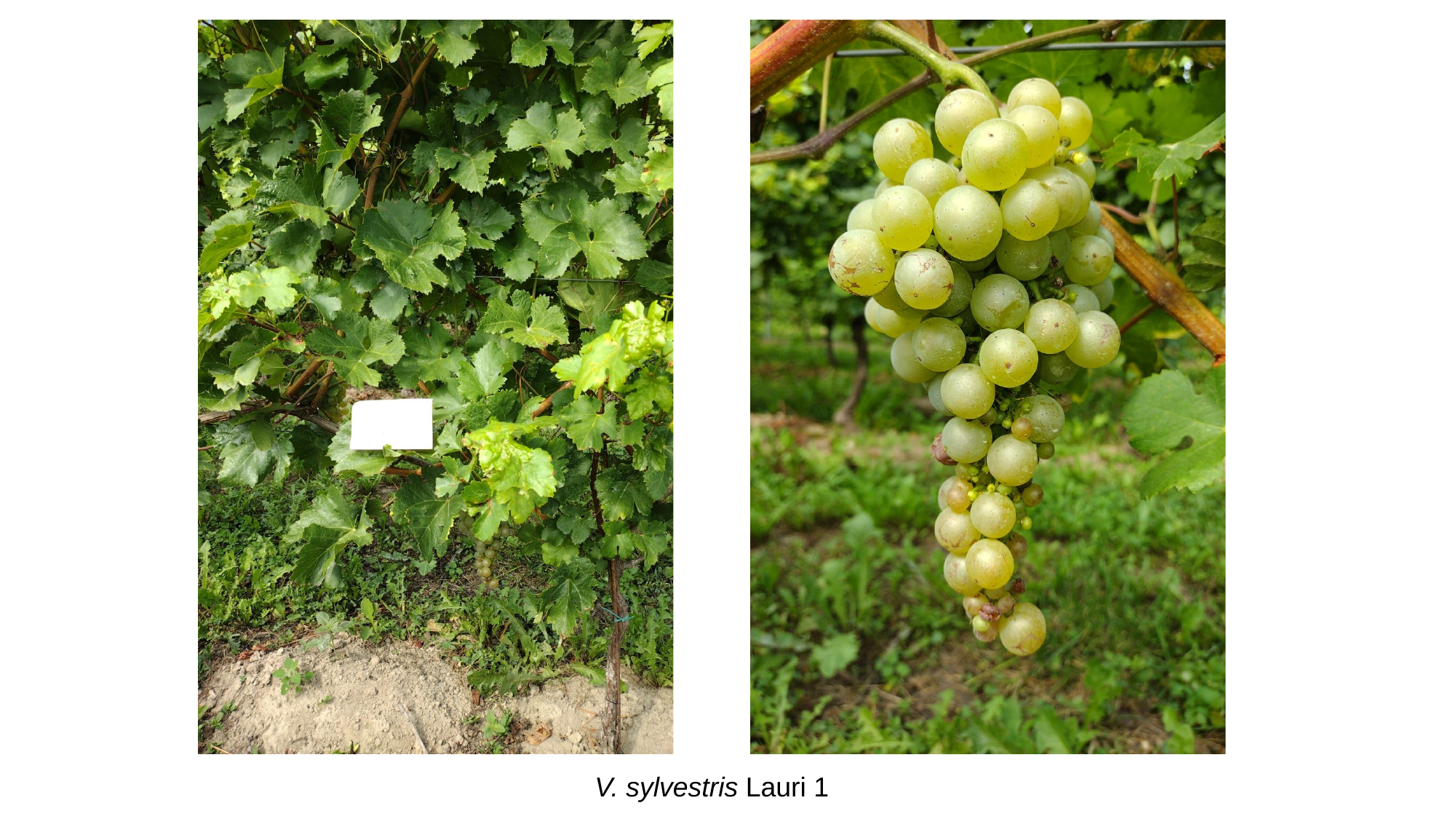

V. sylvestris Lauri 1

Supplement: Supplementary Figure 1 — Principal Component Analysis (PCA) of pairwise genetic relatedness showing the distribution of relationship categories. The first two principal components (PC1 and PC2) explain 64.9% and 23.1% of the total variance, respectively. Points represent pairwise comparisons classified as parent-offspring (PO), full-sibling (FS), half-sibling (HS), grandparent-grandchild (GP), or unrelated (UN). Colored ellipses indicate 95% confidence intervals for each relationship group. The ordination reveals clear separation between close relationships (PO, FS) and more distant or unrelated pairs (HS, GP, UN). [file DataSheet1.zip › Data Sheet/Sup. Figure 4.pptx]

## Slide 1
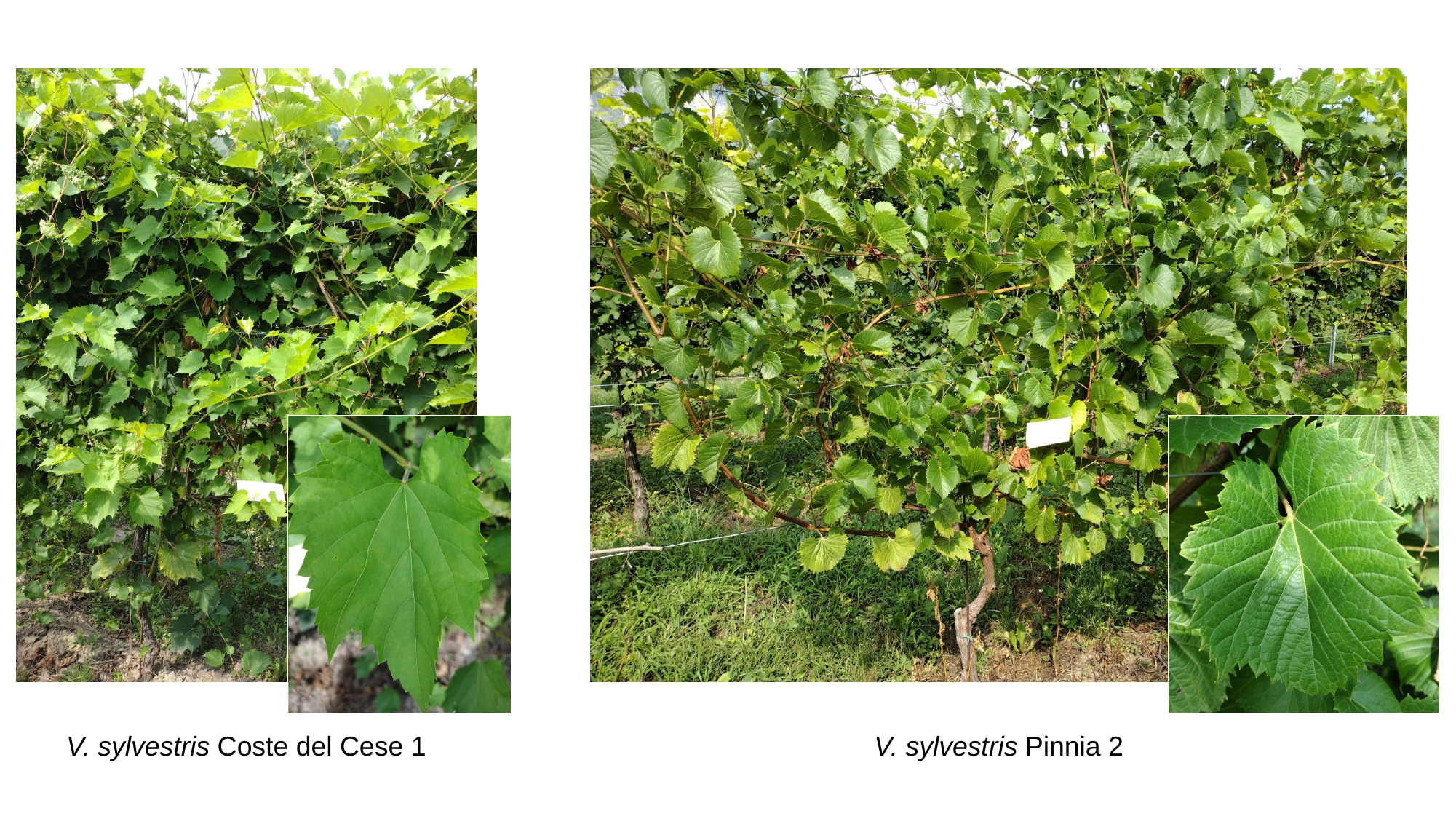

V. sylvestris Coste del Cese 1
V. sylvestris Pinnia 2

Supplement: Supplementary Figure 1 — Principal Component Analysis (PCA) of pairwise genetic relatedness showing the distribution of relationship categories. The first two principal components (PC1 and PC2) explain 64.9% and 23.1% of the total variance, respectively. Points represent pairwise comparisons classified as parent-offspring (PO), full-sibling (FS), half-sibling (HS), grandparent-grandchild (GP), or unrelated (UN). Colored ellipses indicate 95% confidence intervals for each relationship group. The ordination reveals clear separation between close relationships (PO, FS) and more distant or unrelated pairs (HS, GP, UN). [file DataSheet1.zip › Data Sheet/Sup. Figure 3.pptx]
